# Patient-reported outcomes in young adults with osteonecrosis secondary to developmental dysplasia of the hip - a longitudinal and cross-sectional evaluation

**DOI:** 10.1186/s12891-020-03865-3

**Published:** 2021-01-07

**Authors:** Avi Marks, Mario Cortina-Borja, Dror Maor, Aresh Hashemi-Nejad, Andreas Roposch

**Affiliations:** 1 UCL Institute of Child Health, 30 Guilford Street, London, WC1N 1EH UK; 2 Royal National Orthopaedic Hospital, Brockley Hill, Stanmore, HA7 4LP UK

**Keywords:** Developmental dysplasia of the hip, Osteonecrosis, Outcome measures

## Abstract

**Background:**

Osteonecrosis of the femoral head is a common complication in the treatment of developmental dysplasia of the hip (DDH). While functional outcomes of affected patients are good in childhood, it is not clear how they change during the transition to young adulthood. This study determined the relationship between osteonecrosis and hip function, physical function and health status in adolescents and young adults.

**Methods:**

We performed a cross-sectional study of 169 patients with a mean age of 19.7 ± 3.8 years with and without osteonecrosis following an open or closed reduction (1995–2005). We also performed a separate longitudinal evaluation of an historical cohort of 54 patients with osteonecrosis, embedded in this sample. All completed patient-reported outcome measures in 2015/2016 to quantify hip function (maximum score 100); physical function (maximum score 100); and general health status (maximum score 1). We graded all radiographs for subtype of osteonecrosis (Bucholz-Ogden); acetabular dysplasia (centre-edge angle); subluxation (Shenton’s line); and osteoarthritis (Kellgren-Lawrence). Analyses were adjusted for the number of previous surgical procedures on the hip and for the severity of residual hip dysplasia.

**Results:**

In 149 patients (186 hips) with and without osteonecrosis, the mean differences (95% confidence interval) in hip function, physical function and quality of life were − 4.7 (− 10.26, 0.81), − 1.03 (− 9.29, 7.23) and 0.10 (− 1.15, 1.18), respectively. Adjusted analyses stratified across types of osteonecrosis showed that only patients with Bucholz-Odgen grade III had reduced hip function (*p* < 0.01) and physical function (*p* < 0.05) but no difference in health-related quality of life when compared to no osteonecrosis.

**Conclusion:**

Osteonecrosis secondary to DDH is a relatively benign disorder in adolescents and young adulthood. Affected patients demonstrated minimal physical disability, a normal quality of life but reduced hip function.

## Background

Osteonecrosis of the femoral head, also known as physeal arrest, is a serious and frequent complication in the treatment of developmental dysplasia of the hip (DDH) [1–3]. It occurs in up to 73% of closed or open reductions [1, 4–8]. In the absence of curative treatment, osteonecrosis will typically lead to progressive deformity of the hip, associated decline in hip function, disability, pain, and premature osteoarthritis [9, 10].

Osteonecrosis is diagnosed by means of radiography, with the classification schemes by Bucholz-Ogden [11] and Kalamchi-MacEwen [1] used most widely. Both schemes describe four distinctive patterns of physeal arrest, resulting in deformity of the upper femur. In previous research we determined the meaning of these four patterns of anatomical abnormality in terms of patient-based outcomes [12]. We demonstrated that at a mean age of 14 years, Bucholz-Ogden grades III and IV were more often associated with reduced hip function and with greater hip pain than grades I and II. However, none of these grades of osteonecrosis was associated with physical disability or with a reduced quality of life. We concluded that the good results were largely explained by the young patient age of 14 years. We suggested that the patients’ function would decline with increasing age, and that another study involving older patients would be needed to substantiate this hypothesis.

Further longitudinal evaluation of our existing cohort combined with cross-sectional investigations in the transition to adulthood would capture functional outcomes in this age group, which are currently unknown. Delineating within-person changes in such outcomes has the potential to clarify determinants of disease progression associated with osteonecrosis secondary to DDH. The aims of the present study were therefore to ascertain the patient-reported outcomes hip function, physical function, and health status in adolescents and young adults with osteonecrosis secondary to DDH; and how patients with osteonecrosis change over time in terms of these outcomes.

## Methods

The local Research Ethics Committee approved this study (REC 14/LO/1267). We obtained written informed consent from all participants. Eligible for this study were patients with a diagnosis of DDH who had received a closed or open reduction with or without osteotomy and who were older than 14 years of age at the time of study assessment. An attempt was made to include as many patients as possible from our original study [12] as this would allow for inferences to be made about how patients change over time. One researcher (A.M.) used clinical coding and the database from our previous study [12] to identify eligible patients treated in two tertiary centres from 1995 to 2005. We excluded patients with co-morbidities that exclude the diagnosis of DDH.

**Fig. 1:**
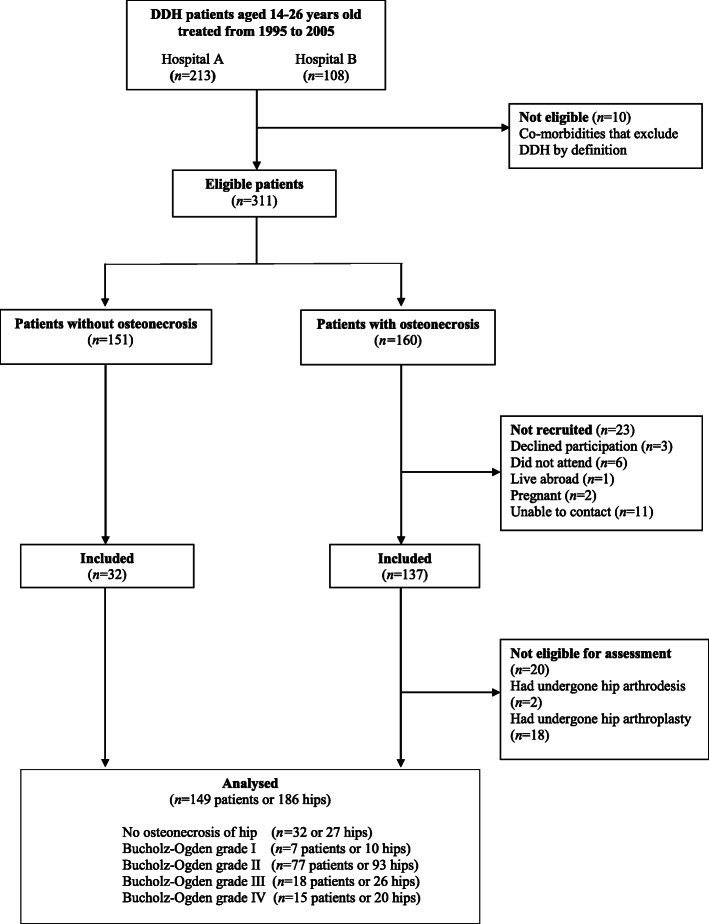
Flow diagram demonstrating sample selection

Of 311 eligible patients identified, 160 (51%) had evidence of osteonecrosis as per clinical records and radiography (Fig. 1). These included 72 patients studied in 2011 [12] when we had measured their hip function, physical function, and health status.

Of 160 patients, 23 could not be recruited (Fig. 1). In 20 of the remaining patients we could not ascertain the effects of the osteonecrosis on patient-reported outcomes as 18 had undergone hip arthroplasties and 2 had undergone hip arthrodesis. Thus, 117 patients (149 hips) with DDH and osteonecrosis at a mean age of 19.6 ± 3.8 years completed patient reported outcomes to measure the effects of osteonecrosis. These included 54/72 patients (75%) who had taken part in our earlier study [12] and who we could re-examine after a mean period (and standard deviation) of 8.4 ± 0.7 years or at a mean patient age (and standard deviation) of 21.9 ± 2.6 years (of the remaining patients, five had undergone total hip replacements; one patient lived abroad; one was pregnant; one had a mental health condition preventing participation; five could not be contacted; and five declined participation). From 151 patients with DDH but without evidence of osteonecrosis, we recruited 32 patients (37 hips) with similar baseline characteristics such as age, sex and the operations received for DDH in childhood. In patients with osteonecrosis 36% of had undergone one operation, 36% two operations, and 28% three or more operations. This compared to 59, 29 and 4% respectively, in patients without osteonecrosis. We thus studied 149 patients (186 hips) at a mean age (and standard deviation) of 19.6 ± 3.8 (range, 14 to 26) years.

All participants had a standing antero-posterior radiograph of the pelvis at the time of study assessment. We employed a standard protocol [13] using a digital imaging system (GE Medical Systems Ltd., Buckinghamshire, UK). We graded the presence of osteonecrosis according to Bucholz-Ogden [11]. In grade I the femoral head shows hypoplasia compared to the unaffected side. In grade II, the lateral growth plate is damaged, resulting in a valgus deformity. Global damage of the physis is said to underlay grade III, resulting in marked shortening of the femoral neck and marked trochanteric overgrowth. Damage along the medial aspect of the physis causes the varus alignment of the upper femur seen in grade IV. Radiographic images depicting each grade were published in our previous study [12]. Grade I we found in 7 patients (10 hips); grade II was seen in 77 patients (93 hips); 18 patients (26 hips) had grade III changes, and 15 patients (20 hips) had changes of grade IV (Fig. 1). We quantified acetabular dysplasia by means of the centre-edge angle of Wiberg [14] and the acetabular angle of Sharp [15]*.* We evaluated the presence of osteoarthrosis according to Kellgren-Lawrence [16]*.* An orthopaedic resident (A.M.) and an orthopaedic fellow (D.M.) analysed all radiographs electronically (Centricity Enterprise Web V3.0. 2006 GE Medical) and in random order, blinded to patient identifiers and clinical variables. They first reviewed all radiographic classifications schemes and agreed on definitions and landmarks. They then evaluated all radiographs independently and their inter-rater reliability was established. For Sharp and centre edge angles, the interrater reliability was excellent [17] (intra-class correlation coefficient = 0.86); it was moderate [17] for the Kellgren-Lawrence (κ = 0.62) and Bucholz-Ogden (κ = 0.64) classifications. For the latter two indices, the two observers reviewed all radiographs in consensus to establish final grade. Radiographs also were graded separately by the senior author (A.R.) for the presence of osteonecrosis and we resolved any disagreements in consensus.

One investigator (A.M.) examined all patients according to the Children’s Hospital Oakland Hip Evaluation Scale (CHOHES) [18]*,* a valid and reliable hip-specific assessment measure with three domains: pain, hip function, and physical examination. *A m*aximum score of 100 points indicates best hip function. We presented the following patient-reported outcome measures to patients in random order to control for an order effect at the group level of analysis:

### Activity scales for kids (ASK)

This 30-item valid and reliable tool measures physical function [19]. Its maximum score is 100 points, indicating unlimited physical functioning. The ASK is intended for patients up to 17 years of age [20] and in those 17 years or older, we used the *Hip Disability and Osteoarthritis Outcomes Score Physical Function Shortform (HOOS-PS)* that also measures physical function [21]. HOOS-PS elicits activity-related symptoms that patients experience due to a hip pathologies. As with the ASK, this measure fits a unidimensional, interval scaled model [22] with a maximum score of 100 indicating unlimited physical functioning.

### Health utilities index Mark 3 (HUI-3)

HUI-3 is a questionnaire-based method for measuring general health status and health-related quality of life, incorporating 8 attributes (vision, hearing, speech, ambulation, dexterity, emotion, cognition, pain) [23]. HUI-3 scores can be converted into utilities [24]. Utility is defined as the strength of an individual preference for a health state measured under conditions of uncertainty, expressed on a continuous scale from 0 to 1, with 0 representing death and 1 representing perfect health [25]. We used a standard 15-item, English-language version for self-administered, self-assessed two-week health-status assessment.

### Statistical methods

The scores on the outcome instruments were summarized with use of the median value and the interquartile range. A proportion of patients had bilateral hip involvement and we determined the relationship between grades of osteonecrosis (none, I, II, III, IV) and outcome measures with linear mixed-effects regression models [26], while adjusting for age at the time of study assessment [12], the total number of operative procedures on the hip of interest (one.

to three or more than three), and acetabular dysplasia (as determined with the lateral center-edge angle, hip migration percentage, and Sharp index) [27]. We used a backwards stepwise elimination approach [28] and Akaike’s Information Criterion (AIC) [29] to assess goodness of fit. We reported least-squares means for adjusted scores. We hypothesized that, on the average, patients with grade-III and IV hips would have more severe outcome scores.

We determined the interrater reliability using Cohen’s weighted κ [30] or the intraclass correlation coefficient model 2 [31], respectively. We estimated the sample size according to Cohen [32] based on the primary outcome, hip function. Established CHOHES scores [12] of 88, 88, 80 and 78 for Bucholz-Ogden grades I-IV respectively (SD = 10) gave effect sizes (Cohen’s *d*) between 0.2 and 0.8. With α = 0.5 and β = 0.20, we estimated at least 15 patients were needed for each Bucholz-Ogden grade examined. In order to adjust for three variables, at least 105 patients with osteonecrosis were required (15 further patients for each additional variable) [33]. We used the *R Language and Environment for Statistical Computing*, version 3.1 statistical package [34].

## Results

**Fig. 2:**
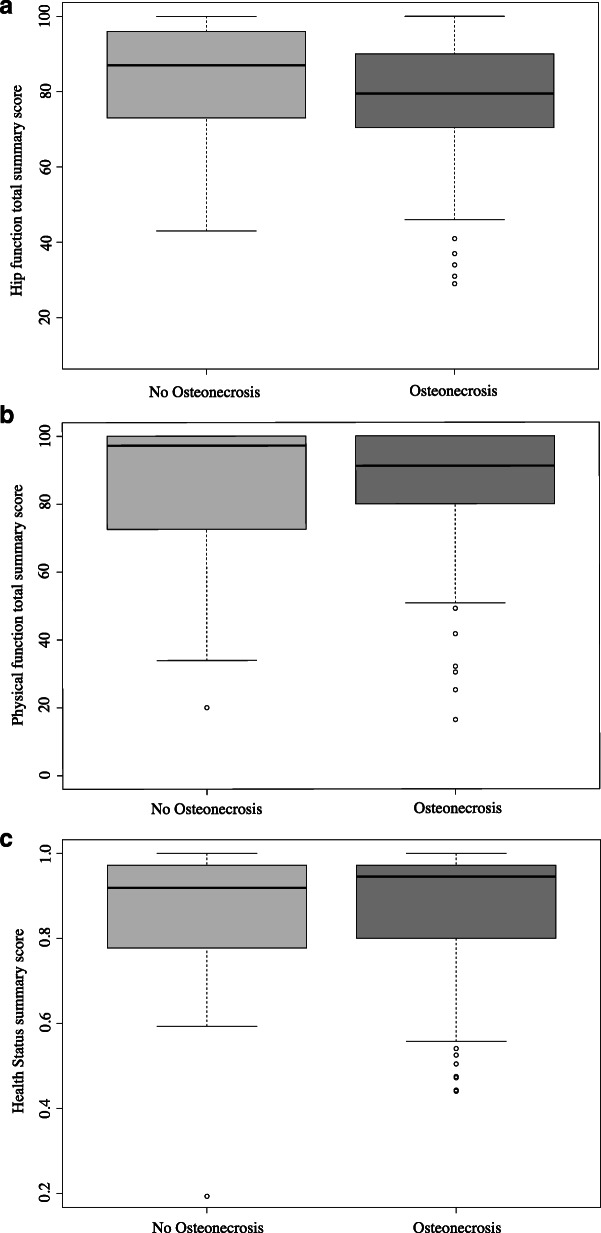
Comparison of hip function (**a**), physical function (**b**), and health status (**c**) scores across grades of osteonecrosis and patients without osteonecrosis. The whisker plots indicate the median and the first and third quartile

In patients with osteonecrosis, the median hip function summary score was 80 (IQR, 70–90); the median physical function score was 91 (IQR, 80–100); and the median health status score was 0.95 (IQR, 0.80–0.97) (Table 1). These scores did not differ (*p* > 0.05) from patients without osteonecrosis (Fig. 2). While the scores of all outcome measures largely declined with increasing Bucholz-Ogden grades, the differences were small and statistically not significant (Table 2). The hip function summary score was nearly equal across all grades of osteonecrosis – adjusted median scores were > 90, indicating normal hip function (Table 3). Subgroup analyses showed hip function differed in those with osteonecrosis grades III/IV when compared with no osteonecrosis (*p* < 0.01) but not when compared with grades I/II (*p* = 0.05). On the ‘hip pain’ and ‘hip function’ subscales, scores did not differ across the five subgroups (*p* > 0.05). However, the ‘physical examination’ subscale showed a reduced score for osteonecrosis grade IV (p < 0.01) (Table 3).

**Table 1:** Distribution of outcome scores according to Bucholz-Ogden grades I to IV in 149 patients. Values represent the median, with the interquartile range in parentheses

Outcome measure	Grade I	Grade II	Grade III	Grade IV	No. of patients with max. Score	No. of patients with min. Score
*Summary scores*						
Hip function	86 (84 to 95)	81 (69 to 94)	78 (70 to 88)	77 (72 to 85)	3 (3%)	2 (2%)
Physical function	91 (82 to 98)	82 (66 to 100)	67.9 (59 to 100)	99 (82 to 100)	33 (28%)	1 (1%)
Health status	0.97 (0.95 to 0.97)	0.95 (0.78 to 0.97)	0.84 (0.74 to 0.97)	0.94 (0.80 to 0.97)	18 (16%)	1 (1%)
*CHOHES domains*						
Hip pain	30 (30 to 40)	30 (30 to 40)	30 (30 to 40)	28 (30 to 40)	44 (38%)	7 (6%)
Hip function	30 (28 to 32)	28 (22 to 31)	26 (22 to 30)	29 (21 to 30)	25 (22%)	1 (1%)
Physical examination	26 (24 to 26)	24 (2 to 25)	20 (17 to 24)	18 (14 to 22)	4 (38%)	1 (1%)
*HUI-3 domains*						
Vision	1 (0.95 to 1)	1 (0.95 to 1)	1 (0.95 to 1)	1 (0.95 to 1)	80 (72%)	30 (27%)
Hearing	1 (1 to 1)	1 (1 to 1)	1 (1 to 1)	1 (1 to 1)	110 (100%)	0
Speech	1 (1 to 1)	1 (0.95 to 1)	1 (1 to 1)	1 (1 to 1)	109 (99%)	1 (1%)
Ambulation	1 (0.73 to 1)	1 (0.73 to 1)	1 (0.73 to 1)	1 (0.73 to 1)	85 (77%)	7 (6%)
Dexterity	1 (0.95 to 1)	1 (0.95 to 1)	1 (1 to 1)	1 (1 to 1)	108 (98%)	2 (2%)
Emotion	1 (0.59 to 1)	1 (0.59 to 1)	1 (0.38 to 1)	1 (0.73 to 1)	68 (62%)	1 (1%)
Cognition	1 (0.38 to 1)	1 (0.38 to 1)	1 (0.59 to 1)	1 (1 to 1)	92 (84%)	8 (7%)
Pain	1 (0.59 to 1)	1 (1 to 1)	1 (1 to 1)	1 (1 to 1)	29 (26%)	4 (4%)

**Table 2:** Mean scores adjusted for age at study assessment, acetabular dysplasia, and number of operations. Values are expressed as the mean and the standard error of the scale points. The maximum possible points for each scale are shown in parentheses

Osteonecrosis	Health status (max. 1)	Physical function (max. 100)	Hip function			
			Summary (max. 100)	Hip pain (max. 40)	Hip function (max. 32)	Physical examination (max. 28)
No Osteonecrosis	0.78 ± 0.14	90.69 ± 17.05	100.00 ± 10.0	40.00 ± 5.73	28.00 ± 3.04	28.46 ± 2.74
Bucholz-Ogden I	0.80 ± 0.07	85.91 ± 8.58	99.48 ± 4.57	38.37 ± 2.88	29.30 ± 0.92	28.82 ± 1.25
Bucholz-Ogden II	0.81 ± 0.04	92.55 ± 4.68	99.07 ± 2.71	40.00 ± 1.65	28.45 ± 0.58	27.66 ± 0.74
Bucholz-Ogden III	0.82 ± 0.05	87.07 ± 6.66	96.83 ± 3.71	39.56 ± 2.24	28.78 ± 0.82	26.66 ± 1.02
Bucholz-Ogden IV	0.78 ± 0.06	85.95 ± 6.84	92.96 ± 3.80	36.63 ± 2.37	28.73 ± 0.81	25.68 ± 1.04

**Table 3:** Changes in patient-reported outcome summary scores in 54 patients who we assessed on two occasions over a mean period of 8.4 years. Values are expressed as the differences in mean scores with 95% confidence intervals, adjusted for age at most recent assessment, number of hip operations per patient, and residual acetabular dysplasia

Bucholz-Ogden grade	Hip function	Physical function	Health status
I	−11.2 (−22.2, −0.1)	−20.5 (−38.6, −2.5)	−0.10 (−0.0, 0.4)
II	8.9 (−17.0, 34.8)	7.2 (−8.9, 30.1)	− 0.06 (− 0.3, 0.4)
III	8.3 (−19.3, 35.9)	6.2 (− 20.9, 23.7)	− 0.07 (− 0.4, 0.3)
IV	−11.3 (−41.2, 18.6)	4.6 (−30.4, 19.5)	−0.11 (− 0.4, 0.6)

We found no difference in physical function or health status between patients with and without osteonecrosis. These outcomes were equally similar (p > 0.05) across all Bucholz-Ogden grades (Table 3).

In 54/72 patients (75%) with longitudinal data for outcome scores available, the mean within- patient changes in hip function, physical function and health status from baseline to current assessment were 7.18 (95% confidence interval, − 2.11 to 12.26), − 2.11 (95% confidence interval, − 15.47 to 11.25), and − 0.03 (95% confidence interval, − 0.11 to 0.05), respectively. While these differences indicate that patients did not change within 8 years of follow-up in those three outcomes, five of 72 patients (6%) had required hip arthroplasty.

## Discussion

Postoperative complications have been identified as poor prognostic factors for hip survivorship, the most serious being osteonecrosis of the proximal femoral epiphysis. This has been reported to have occurred in 60% of patients following closed reduction [9]. However, there is considerable variation in the reported incidence, ranging from 0 to 73% [4–8] following closed or open reduction, which is likely to reflect the inconsistency in diagnostic ability. The external validity of the osteonecrosis rates are further undermined by the heterogeneity in the interventions preceding diagnosis, different case definitions, inconsistencies in the outcome measures and variable participant attrition rates. Many studies lack adequate length of follow-up to appreciate radiological signs that may appear after many years [35].

While the precise mechanism of osteonecrosis is not known, its iatrogenic nature is not disputed as it is not seen in the absence of treatment [9]. Patho-morphological changes of the proximal femur ultimately result in a variable growth disturbance of the proximal femoral physis which can manifest as a lack of femoral head sphericity, shortening and deformity of the femoral neck, abnormality of the metaphyseal region, and overgrowth of the greater trochanter with resultant abductor musculature weakness [36].

Risk factors for osteonecrosis highlighted in a number of long-term studies include the patient’s age at surgery [37], perioperative injury to the proximal femoral blood supply [38], and an eccentric position of the femoral head in plaster [39].

Osteonecrosis can be devastating as it leads to deformities to the femoral head, with changes in the proximal femoral physis key to predicting residual deformity [1]. Previous work demonstrated osteonecrosis compromises subsequent acetabular remodelling thereby causing secondary abnormalities to the acetabulum [2]. Other deformities resulting from osteonecrosis that are associated with osteoarthritis include shape mismatch in the convexity of the femoral head and concavity of the acetabulum, persistent lateral and proximal subluxation, and medial femoral head irregularity [6].

Treatments for osteonecrosis concentrate on improving the biomechanics of the hip, as interventions directly addressing early proximal femoral growth disturbances by repairing physeal injuries or altering the blood supply to the physis are not yet available.

Understanding this complication is so important as children and adolescents with this irreversible condition experience early decline in function, pain and premature osteoarthritis; and many patients with ‘severe’ osteonecrosis require hip arthroplasty from late adolescence [40]. Even hips with minor anatomical abnormalities and residual deformity are at risk of premature osteoarthritis because of DDH [41].

Our previous research showed that the effects of osteonecrosis were benign in childhood: at a mean age of 14 years affected patients demonstrated nearly normal physical functioning, a normal health-related quality of life, and minor limitations of hip function [12]. The present study examined whether this remained the case in adolescence and in young adulthood. To do this, we performed a cross-sectional evaluation of patients at a mean age of 20 years, as well as a longitudinal assessment of 54 patients with a mean age of 22 years who we had studied in 2011.

Changes associated with osteonecrosis (physeal arrest) involve the proximal femur and this could impair hip function. We previously showed that at a mean age of 14 years hip function was minimally affected by osteonecrosis, ranging from 94/100 points on the CHOHES for grade I osteonecrosis, to 78/100 points in grade IV [12]. This study confirmed that, at a mean age of 21 years, it ranged from 86/100 points to 77/100 points. Aguilar et al. [18], using the CHOHES, found a mean score of 88/100 points in children without any hip problems. This would indicate that the hip function of our young adult patients was reduced in those with osteonecrosis grades III and IV by a degree that was clinically important. However, these differences were no longer seen in the adjusted analysis – scores were above 88/100 points and almost identical across all four grades of osteonecrosis. This suggests that the effects of the osteonecrosis alone did not explain the reduced hip function in grades III and IV. When assessing the CHOHES subscales, ‘physical examination’ scores declined with increasing Bucholz-Ogden grades (Table 3) indicating reduced range of motion in radiographically more severely affected hips. Overall, the isolated effects of the radiographic changes associated with physeal arrest were small in terms of hip-specific function.

In terms of physical function and general health status, the adolescent and young adult patients did well, but those with grade III osteonecrosis showed lower scores than all other grades of osteonecrosis (Table 2). But again, these differences were no longer seen in the adjusted analyses suggesting that regardless of the grade of osteonecrosis, these patients had near normal scores for physical function and for general health status. This is similar to what was found in children at a mean age of 14 years [12], and with an analysis of normal individuals where ASK scores as low as 80 have been observed in nondisabled children [42].

From the adjusted analysis it appears that the drivers for low patient-reported outcome scores were not the grades of osteonecrosis, but other factors. Residual acetabular dysplasia is generally associated with less favourable functional outcome scores [9]. Persistent subluxation leads to a reduced contact surface between femoral head and acetabulum, thus increasing the risk for osteoarthritis [43]. Three of the lowest CHOHES scores (< 45) in this study were encountered in patients with grade IV osteonecrosis *with* marked acetabular dysplasia and subluxation. A further three patients with a CHOHES score < 45 showed grade III osteonecrosis; all had bilateral DDH and osteoarthritic changes of grades III and IV according to Kellgren-Lawrence [4]. Our comparison group of 32 patients who underwent childhood treatment of DDH further demonstrated that factors other than osteonecrosis drove low outcome scores: 43% of their hips scored less than 85/100 on the CHOHES despite the absence osteonecrosis. Of these, three hips showed a centre-edge angle < 20 degrees; three hips had Kellgren-Lawrence grade 0; and six hips had Kellgren-Lawrence grade 1.

In this study we were able to report, for the first time, how patients with osteonecrosis and DDH changed over eight years by re-examining 54 of 72 patients (75%) who took part in a previous study [12]. These changes were minimal, suggesting that patients maintained high levels of hip function, physical function and health status if their hip survived. However, 5 of 72 patients (6%) had needed a hip replacement within this time period, indicating they had severe hip-related disabilities. It is unknown how the remaining 13 patients not included in this follow up study faired over those eight years – this limits our conclusions about how patients change over time. Yet, analysis of the baseline variables does not reveal systematic differences between cohort patients who were or were not recruited for follow-up.

We note other potential limitations of this study. The participants of this study may have been too young to discern the ultimate effects of osteonecrosis on patient-reported outcomes. However, we selected this age group deliberately to gather insight into patients transitioning from paediatric to adult health care services, when activities and demands change after leaving school [44] often heralding the onset of functional impairments [6]. We utilised two different instruments for measuring ‘physical function’ based on patient age (80% used the HOOS-PS and 20% used the ASK). However, we think this was acceptable because factor analysis showed [45] that items of the ASK loaded on two distinct factors, ‘activities of daily living’ and ‘play/sport’, which are also the underlying constructs of the HOOS-PS. Both instruments have a very similar method of scoring, but as we could not assume a normal distribution for these response variables (the scores tended to be skewed towards better function), we used quantile regression to establish equivalence of scores.

Gibson and Benson [46] recognised that following treatment of developmental dysplasia of the hip, poor radiographic appearances of the hip may cause few symptoms until late adolescence or early adult life. By re-assessing patients from our original cohort and by assessing newly recruited patients of a similar age, we sought to further evaluate disease severity associated with osteonecrosis in patients transitioning from paediatric to adult care.

## Conclusion

We demonstrated that at a mean age of 21 years, patients with and without osteonecrosis, on the whole, reported high scores in patient-reported outcomes. There are outliers with poor functional scores, but it would appear that factors other than osteonecrosis contribute to this disability. Clinicians could use this information when counselling the carers of affected children early when a diagnosis of osteonecrosis is made. It may also aid in determining the need (or frequency) for orthopaedic follow up appointments after skeletal maturity. Our future research will delineate which distinct radiographic features of osteonecrosis are likely to lead to a rapid decline in functional outcomes.

## Data Availability

The datasets used and/or analysed during the current study are available from the corresponding author on reasonable request.

## Abbreviations

DDH: developmental dysplasia of the hip
CHOHES: Children’s Hospital Oakland Hip Evaluation Scale
ASK: Activity Scales for Kids
HOOS-PS: Osteoarthritis Outcomes Score Physical Function Shortform
HUI-3: Health Utilities Index Mark-3
